# TNFAIP3 Gene Polymorphisms in Three Common Autoimmune Diseases: Systemic Lupus Erythematosus, Rheumatoid Arthritis, and Primary Sjogren Syndrome—Association with Disease Susceptibility and Clinical Phenotypes in Italian Patients

**DOI:** 10.1155/2019/6728694

**Published:** 2019-08-27

**Authors:** C. Ciccacci, A. Latini, C. Perricone, P. Conigliaro, S. Colafrancesco, F. Ceccarelli, R. Priori, F. Conti, R. Perricone, G. Novelli, P. Borgiani

**Affiliations:** ^1^UniCamillus, Saint Camillus International University of Health Sciences, Rome, Italy; ^2^Department of Biomedicine and Prevention, Genetics Section, University of Rome Tor Vergata, Italy; ^3^UOC Reumatologia, Dipartimento di Medicina Interna e Specialità Mediche, Sapienza Università di Roma, Italy; ^4^Clinic of Rheumatology, Allergology and Clinical Immunology, Department of Medicina dei Sistemi, University of Rome Tor Vergata, Italy

## Abstract

Autoimmune diseases (AIDs) are complex diseases characterized by persistent or recurrent inflammation, alteration of immune response, and production of specific autoantibodies. It is known that different AIDs share several susceptibility genetic loci. Tumor necrosis factor alpha inducible protein 3 (TNFAIP3) encodes the ubiquitin-modifying enzyme A20, which downregulates inflammation by restricting NF-*κ*B, a transcription factor that regulates expression of various proinflammatory genes. Variants in *TNFAIP3* gene have been described as associated with susceptibility to several AIDs. Here, we analyzed two *TNFAIP3* polymorphisms in Italian patients with systemic lupus erythematosus (SLE), rheumatoid arthritis (RA), and primary Sjogren's syndrome (pSS), to verify if the genetic variability of *TNFAIP3* gene is involved in genetic predisposition to AIDs also in the Italian population. We recruited 313 SLE patients, 256 RA patients, 195 pSS patients, and 236 healthy controls. Genotyping of rs2230926 and rs6920220 in *TNFAIP3* gene was performed by an allelic discrimination assay. We carried out a case/control association study and a genotype/phenotype correlation analysis. A higher risk to develop SLE was observed for rs2230926 (*P* = 0.02, OR = 1.92). No association was observed between this SNP and the susceptibility to pSS or RA. However, the rs2230926 variant allele seems to confer a higher risk to develop lymphoma in pSS patients, while in RA patients, the presence of RF resulted significantly associated with the variant allele. Regarding the rs6920220 SNP, we observed a significant association of the variant allele with SLE (*P* = 0.03, OR = 1.53), pSS (*P* = 0.016, OR = 1.69), and RA (*P* = 0.0001, OR = 2.35) susceptibility. Furthermore, SLE patients carrying the variant allele showed a higher risk to develop pericarditis, pleurisy, and kidney complications. Our results support the importance of the *TNFAIP3* gene variant role in the development of different autoimmune diseases in the Italian population and furtherly confirm a sharing of genetic predisposing factors among these three pathologies.

## 1. Introduction

Systemic lupus erythematosus (SLE), rheumatoid arthritis (RA), and primary Sjogren syndrome (pSS) are complex autoimmune diseases characterized by the alteration of the immune response and by inflammation. The aetiology of these disorders is only partially known, but it is nowadays recognized that several factors, including genetics and environment, are involved in their development. In the last decade, due to the use of new genomic technologies, many genetic factors have been identified as associated with the susceptibility to different autoimmune diseases. In a particular way, genome-wide association studies (GWAS) brought a great contribution to the identification of new genetic risk factors for these diseases [1–6]. Among the three diseases, SLE and RA have been the most investigated regarding their genetic basis, and more than 100 different loci have been confirmed as associated with their susceptibility [7, 8]. Up to now, the number of loci associated to pSS is considerably lower [9].

Since these autoimmune diseases share some clinical characteristics, it is not surprising that they also share many of the associated loci [10–12]. Among the shared risk loci, one of the most confirmed is the tumor necrosis factor (TNF) alpha-induced protein 3 (*TNFAIP3*) gene. Candidate gene studies and GWAS have reported associations of *TNFAIP3* polymorphisms with several autoimmune diseases, including also SLE, RA, and pSS [10, 13–15]. *TNFAIP3* codes for a protein, called A20, which is a negative regulator of NF-*κ*b. Its deficiency in immune cells has been linked to inflammation and autoimmunity [16, 17]. Expression studies have reported a lower level of TNFAIP3 in patients with SLE compared with healthy controls [18].

In the present study, we investigated two *TNFAIP3* gene SNPs in order to verify their possible association with the susceptibility to three different autoimmune diseases, specifically SLE, RA, and pSS, in Italian patients. We also evaluated the contribution of these genetic variants with respect to different clinical phenotypes of these diseases.

## 2. Materials and Methods

### 2.1. Patients' Recruitment

Patients investigated in this study were previously described [19–21]. We included 313 SLE patients, 256 RA patients, 195 pSS patients, and 236 healthy controls. SLE patients were diagnosed according to the revised 1997 American College of Rheumatology criteria and retrospectively recruited at the Lupus Clinic of the Rheumatology Unit (Sapienza, University of Rome). RA patients, which diagnoses were made according to the 2010 American College of Rheumatology (ACR)/European League Against Rheumatism (EULAR) classification criteria, were retrospectively recruited at the Rheumatology Outpatient Clinic at the Department of “Medicina dei Sistemi” (Policlinico Tor Vergata, Rome, Italy). The pSS patients were diagnosed according to the American European Consensus Criteria and enrolled at Sjӧgren's Clinic of the Rheumatology Unit (Sapienza, University of Rome). A complete description of demographic and clinical characteristics of patients is reported in Supplementary Materials (Tables [Supplementary-material supplementary-material-1]–[Supplementary-material supplementary-material-1]). All patients signed the written informed consent. The study protocol was approved by the local ethics committee of the Sapienza University of Rome for SLE and pSS samples and University of Rome “Tor Vergata” for RA samples.

### 2.2. DNA Extraction and Genotyping

Genomic DNA was isolated from peripheral blood mononuclear cells using a Qiagen blood DNA mini kit (Qiagen, Valencia, VA, USA). All subjects were investigated for the rs2230926 and the rs6920220 *TNFAIP3* polymorphisms by an allelic discrimination assay by TaqMan assays (Applied Biosystems, Foster City, CA, USA) and ABI PRISM 7500. We always used samples with known genotypes (detected by direct sequencing) in each run of the allelic discrimination assay as genotype controls.

### 2.3. Statistical Analysis

The Hardy-Weinberg equilibrium as well as the differences in the alleles and genotype distribution was evaluated by the Pearson *χ*^2^ test. Differences in genotype frequencies between groups of patients were evaluated by the Pearson *χ*^2^ test or by Fisher's exact test, where appropriate. Odds ratios (ORs) with 95% CI were calculated. Haplotypes between the two SNPs were inferred by Haploview, version 4.2. Differences in haplotype distribution between cases and controls were evaluated by the Chi-square test. Two-tailed *P* values less than 0.05 were considered statistically significant. Statistical analyses were performed by the SPSS program ver. 19 (IBM Corp., Armonk, NY, USA).

## 3. Results

764 Italian AD patients were included in the study, comprising 313 SLE patients, 256 RA patients, and 195 pSS patients. Clinical and demographic characteristics of all patients were described in supplementary Tables [Supplementary-material supplementary-material-1]–[Supplementary-material supplementary-material-1]. Hardy-Weinberg equilibrium has been verified for both investigated SNPs. Results of genotyping and case/control association analysis are reported in Table 1. As shown, the variant allele of rs2230926 SNP was associated only with SLE susceptibility both at genotypic (*P* = 0.02 and OR = 1.92) and allelic (*P* = 0.15 and OR = 1.91) levels. No trend for association was observed for pSS and RA. With regard to the rs6920220 SNP, the variant allele was associated with the susceptibility to SLE (*P* = 0.03, OR = 1.53), RA (*P* < 0.0001, OR = 2.35), and pSS (*P* = 0.016, OR = 1.69) at the genotypic level. The association was maintained also at the allelic level for RA (*P* < 0.0001 and OR = 1.99) and for pSS (*P* = 0.02 and OR = 1.55), respectively.

The comparison of haplotype (considering the two SNPs) distributions between cases and controls did not improve the statistical significance with respect to the single SNP associations (data not shown).

A genotype/phenotype correlation analysis was performed revealing significant associations with some clinical phenotypes. Although not associated with RA or pSS susceptibility, the variant allele of rs2230926 SNP seems to confer a higher risk to develop lymphoma (*P* = 0.001, OR = 9.5) in pSS patients, and it was more present in rheumatoid factor-positive RA patients (*P* = 0.034, OR = 3.1) (Table 2).

On the contrary, the rs6920220 SNP showed correlations only with SLE clinical phenotypes: the variant allele seems to confer a higher risk to develop pericarditis (*P* = 0.007, OR = 2.25), pleurisy (*P* = 0.033, OR = 2.12), and nephritis (*P* = 0.0004, OR = 2.48) but also confers a protective effect with respect to the reduction of C4 (*P* = 0.012, OR = 0.50) and C3 (*P* = 0.026, OR = 0.55) levels and with respect to the development of thrombocytopenia (*P* = 0.017, OR = 0.36) (Table 3).

## 4. Discussion

SLE, RA, and pSS are inflammatory rheumatic diseases with the involvement of the immune system and systemic manifestations. Although they are different in frequency, severity, and clinical characteristics, they all can evolve in a condition of chronicity and severity, often causing an important disability for the affected patient. Their prompt diagnosis is important to start the appropriate treatment and to avoid anatomical damage that could reduce quality and life expectancy in most patients.

These diseases show various clinical similarities, including the presence of autoantibodies, the possible onset of common phenotypes, such as leukopenia, myalgia, and arthralgia/arthritis, and a higher prevalence in female sex. Given these common features, it is not surprising that they can also share part of the genetic susceptibility component. Specifically, in this study, we investigated the role of *TNFAIP3* polymorphisms in Italian patients affected by the three different diseases, each one represented by a well-characterized cohort of patients. Indeed, although there are several studies on *TNFAIP3* and even meta-analysis [6] regarding the association of this gene in many diseases, it has not been investigated in the Italian patients with autoimmune diseases.

Our results showed that the gene, or at least the investigated SNPs, are associated also in our population of study. Specifically, the rs2230926 was associated only with SLE susceptibility, while rs6920220 was associated with all three diseases. These findings, in terms of disease susceptibility, are in line with data in literature and with results obtained in different populations [22–24]. Moreover, a recent meta-analysis confirmed the association of G allele of rs2230926 with an increased risk of SLE in Caucasians, Asians, and Africans [25]. Since, among the five Caucasian samples included in this meta-analysis, only one was recruited in Europe (specifically in Sweden), our study is a confirmation that this variant associates with SLE in European populations.

Nonetheless, the most interesting results of our study come from the genotype-phenotype correlations. Although we did not observe an association between rs2230926 and RA or pSS susceptibility, the variant allele of this SNP seems to correlate with a more severe phenotype in both diseases. In RA patients, the variant allele is associated with RF production, conferring a higher risk to manifest this subphenotype. RF is a well-known negative prognostic factor as recognized by EULAR recommendations for the management of RA [26]. High RF serum levels have been associated with an aggressive articular disease, extra-articular manifestations, and a worse outcome [27].

Similarly, in pSS, the variant allele seems to be predictive of a more aggressive disease, conferring a higher risk to develop lymphoma, practically the most severe complication in pSS. This result is in agreement with the study of Nocturne et al. performed in two independent larger cohorts of pSS from UK and France, in which the SNP was not associated with the disease susceptibility but with a higher risk to develop lymphoma [28]. Although our sample is smaller and the variant allele frequency is lower (both in cases and in controls) than that observed in UK and French cohorts, we can confirm the association of this SNP with the occurrence of lymphoma also in our Italian population.

The variant allele of rs6920220 showed associations only with SLE clinical phenotypes; in particular, it conferred a higher risk to develop clinical features such as pericarditis, pleurisy, and nephritis but a protective role with respect to decreased C3 and C4 levels in the clinical history, suggesting that *TNFAIP3* gene may be involved in different disease pathways [28].

*TNFAIP3* encodes the deubiquitinase A20, and its expression is induced by inflammatory proteins such as IL-17 or TNF-*α* [17]. Indeed, A20 acts as a feedback inhibitor of inflammatory pathways, and its deficiency led to enhanced IL-17-dependent expression of inflammatory genes [29]. IL-17 is the signature cytokine of T helper 17 (TH17), implicated in numerous autoimmune diseases. Many studies had already analyzed genes involved in the TH17 response; for example, one of the most investigated has been the signal transducer and activator of transcription 4 (*STAT4*) gene. Also, our previous studies in the Italian population, in agreement with literature data [30], have confirmed a variant allele located on *STAT4* gene as associated with major risk to develop SLE [31], RA [19], and pSS [21]. These evidences support the already accredited hypothesis that TH17 plays a ubiquitous role in different autoimmune diseases.

The investigated variants were reported as associated also with other different autoimmune diseases. In particular, the variant allele of rs2230926 seems to be more frequent in subjects with polymyositis and dermatomyositis [32], while a strong association of rs6920220 was described with a major risk to develop juvenile idiopathic arthritis [33]. Moreover, further SNPs of TNFAIP3 genes have been observed associated with the RA, SLE, and SS susceptibility [3, 24].

The strength of this study consists in the fact that we investigated different autoimmune diseases from the same Italian population. Moreover, for each disease, the diagnostic assessment was carried out in an accurate and very homogeneous way and according to current guidelines. Admittedly, the sample size of each disease is not very large, but it is sufficient to identify the associations with the diseases and with the clinical phenotypes. Indeed, our patients are very well characterized for clinical and phenotypic features, allowing performing the genotype-phenotype correlation analysis that is not always possible when samples are collected in different centres. This is another point of strength of our study since genetic factors that are not associated with the disease susceptibility could be instead associated with the modulation and the occurrence of specific phenotypes. This aspect should be taken into account for the early identification of patients that are more prone to develop severe phenotypes and to provide a better monitoring of patients.

The identification of common genetic risk factors for different autoimmune diseases may help in stratifying patients, making an early diagnosis and providing a more appropriate treatment. However, even if hundreds of susceptibility genetic loci have been identified so far, and many of them are shared among different diseases, it is important to conduct association studies and replication studies at the population level. Of course, besides the identification of the genetic component, it should be very important to explore the possible role of other factors such as epigenetics, in particular, microRNAs, which could explain part of the susceptibility component not yet identified.

## Figures and Tables

**Table 1:** Distribution of genotypes and alleles of the investigated TNFAIP3 polymorphisms in SLE, pSS, RA, and healthy controls.

		Genotypes					Alleles			
rs2230926 T>G	N	TT	TG	GG	*P* ^a^	OR	T	G	*P*	OR
CTRL	236	217	18	1			452	20		
SLE	313	268	41	4	**0.02**	1.92 (1.09-3.38)	577	49	**0.015**	1.91 (1.12-3.28)
pSS	195	183	11	1	0.45	0.75 (0.35-1.58)	377	13	0.49	0.78 (0.38-1.59)
RA	256	229	26	1	0.23	1.45 (0.79-2.66)	484	28	0.37	1.31 (0.73-2.35)

rs6920220 G>A	N	GG	AG	AA	*P* ^a^	OR	G	A	*P*	OR
CTRL	228	176	45	7			397	59		
SLE	308	212	91	5	**0.03**	1.53 (1.03-2.27)	515	101	0.12	1.32 (0.93-1.87)
pSS	195	130	56	9	**0.016**	1.69 (1.1-2.6)	316	74	**0.02**	1.55 (1.07-2.26)
RA	256	151	93	12	**<0.0001**	2.35 (1.58-3.50)	395	117	**<0.0001**	1.99 (1.42-2.81)

^a^Heterozygous and homozygous variant genotypes were considered together (one degree of freedom) in the comparisons. CTRL: healthy controls; SLE: systemic lupus erythematosus; pSS: primary Sjogren's syndrome; RA: rheumatoid arthritis; OR: odds ratio. Significant associations are reported in bold.

**Table 2:** Association between disease phenotypes and rs2230926 (T>G) polymorphism.

	TT	TG+GG	*P*	OR
Primary Sjogren's syndrome				
Without lymphoma	171	9	**0.001**	9.5 (2.04-44.38)
With lymphoma	6	3		
Rheumatoid arthritis				
RF negative	77	4	**0.034**	3.1 (1.04-9.26)
RF positive	149	24		

OR: odds ratio; RF: rheumatoid factor.

**Table 3:** Association between SLE phenotypes and rs6920220 polymorphism.

	GG	GA+AA	*P*	OR
Without pericarditis	176	70	**0.007**	2.25 (1.24-4.1)
With pericarditis	29	26		
Without pleurisy	151	64	**0.033**	2.12 (1.05-4.28)
With pleurisy	20	18		
Without nephritis	153	52	**0.0004**	2.48 (1.49-4.15)
With nephritis	51	43		
Without thrombocytopenia	135	77	**0.017**	0.36 (0.15-0.85)
With thrombocytopenia	34	7		
Normal C4	76	55	**0.012**	0.50 (0.29-0.86)
Decreased C4	85	31		
Normal C3	66	48	**0.026**	0.55 (0.32-0.93)
Decreased C3	95	38		

OR: odds ratio.

## Data Availability

The data used to support the findings of this study are available from the corresponding author upon request.

## Supplementary Materials

Supplementary Materials Table S1: demographic and clinical characteristics of the 313 SLE patients. Table S2: demographic and clinical characteristics of the 256 RA patients. Table S3: demographic and clinical characteristics of the 195 pSS patients.
