# Supplementary material for: TNFAIP3 Gene Polymorphisms in Three Common Autoimmune Diseases: Systemic Lupus Erythematosus, Rheumatoid Arthritis, and Primary Sjogren Syndrome—Association with Disease Susceptibility and Clinical Phenotypes in Italian Patients
Source: J Immunol Res. 2019 Aug 27;2019:6728694. doi: 10.1155/2019/6728694 (PMC6732636; doi:10.1155/2019/6728694)
Supplement: Supplementary Materials — Table S1: demographic and clinical characteristics of the 313 SLE patients. Table S2: demographic and clinical characteristics of the 256 RA patients. Table S3: demographic and clinical characteristics of the 195 pSS patients. [file 6728694.f1.docx]

| **TableS1. Demographic and clinical characteristics of the 313 SLE patients** | |
| --- | --- |
| Age (years±SD) | 43.06+-11.28 |
| Age at onset (years±SD) | 32.3+-12 |
| Sex (female) | 90.4% |
| Musculo-skeletal involvement | 66.7% |
| Photosensitivity | 63.4% |
| Malar rash | 58.6% |
| Aphthous/ulcers | 20.4% |
| Pericarditis | 17.9% |
| Pleurisy | 14.8% |
| Nephritis | 31% |
| Neuropsychiatric | 13.9% |
| Anemia | 43.7% |
| Leucopoenia | 39.7% |
| Thrombocytopenia | 16.2% |
| ANA | 95.4% |
| Anti-dsDNA | 69.3% |
| Anti-Sm | 13.7% |
| Anti-RNP | 17.8% |
| Anti-Ro/SSA | 32.8% |
| Anti-La/SSB | 14.3% |
| Anti-CL IgG or IgM | 38.1% |
| Anti-β2GPI IgG or IgM | 21% |
| LAC | 28.5% |
| C3 (below normal values) | 53% |
| C4 (below normal values) | 46.2% |

| **TableS2. Demographic and clinical characteristics of the 256 RA patients** | |
| --- | --- |
| Age (years±SD) | 60.9 ± 13.4 |
| Age at diagnosis (years±SD) | 47.52 ± 14.79 |
| Sex (female) | 75.2% |
| Rheumatoid factor | 66.9% |
| ACPA positive | 68.1% |
| DAS28 (means±SD) | 5.16 ± 1.31 |
| Erosions | 60.4% |

| **TableS3. Demographic and clinical characteristics of the 195 pSS patients** | |
| --- | --- |
| Age (years±SD) | 58.31± 11.066 |
| Age at diagnosis (years±SD) | 51.40 ± 11.177 |
| Sex (female) | 94.4% |
| Xerostomia | 89.6% |
| Xerophthalmia | 93.8% |
| Anti-Ro/SSA | 65.5% |
| Anti-La/SSB | 45.5% |
| Anti-nuclear (ANA) | 87.1% |
| Rheumatoid factor | 37.0% |
| Hypergammaglobulinemia | 35.3% |
| Cryoglobulinemia | 4.3% |
| Monoclonal component | 8.7% |
| Hypocomplementemia | 11.4% |
| Leucopenia | 23.0% |
| Lymphopenia | 17.3% |
| Neutropenia | 3.2% |
| Lymphoma | 4.8% |
| Glandular swelling | 22.5% |
| Arthritis | 12.0% |
